# 2-*C*-Cyclo­hexyl-2,3-*O*-isopropyl­idene­erythrofuran­ose

**DOI:** 10.1107/S1600536809048557

**Published:** 2009-11-21

**Authors:** Tony V. Robinson, Dennis K. Taylor, Edward R. T. Tiekink

**Affiliations:** a Discipline of Chemistry, University of Adelaide, 5005 South Australia, Australia; b Discipline of Wine and Horticulture, University of Adelaide, Waite Campus, Glen, Osmond 5064, South Australia, Australia; c Department of Chemistry, University of Malaya, 50603 Kuala Lumpur, Malaysia

## Abstract

In the title compound, C_13_H_22_O_4_, the acetonide ring adopts an envelope conformation with one of the O atoms as the flap atom, whereas a twisted conformation is found for the furan­ose ring. Centrosymmetric eight-membered {⋯OCOH}_2_ synthons involving the hydr­oxy H and acetonide O atoms are found in the crystal structure. These are linked into a supra­molecular chain in the *a*-axis direction *via* C—H⋯O contacts.

## Related literature

For the dihydroxy­lation of the olefin portion of 1,2-dioxines, see: Robinson *et al.* (2006 ▶, 2009 ▶); Valente *et al.* (2009 ▶); Pedersen *et al.* (2009 ▶).
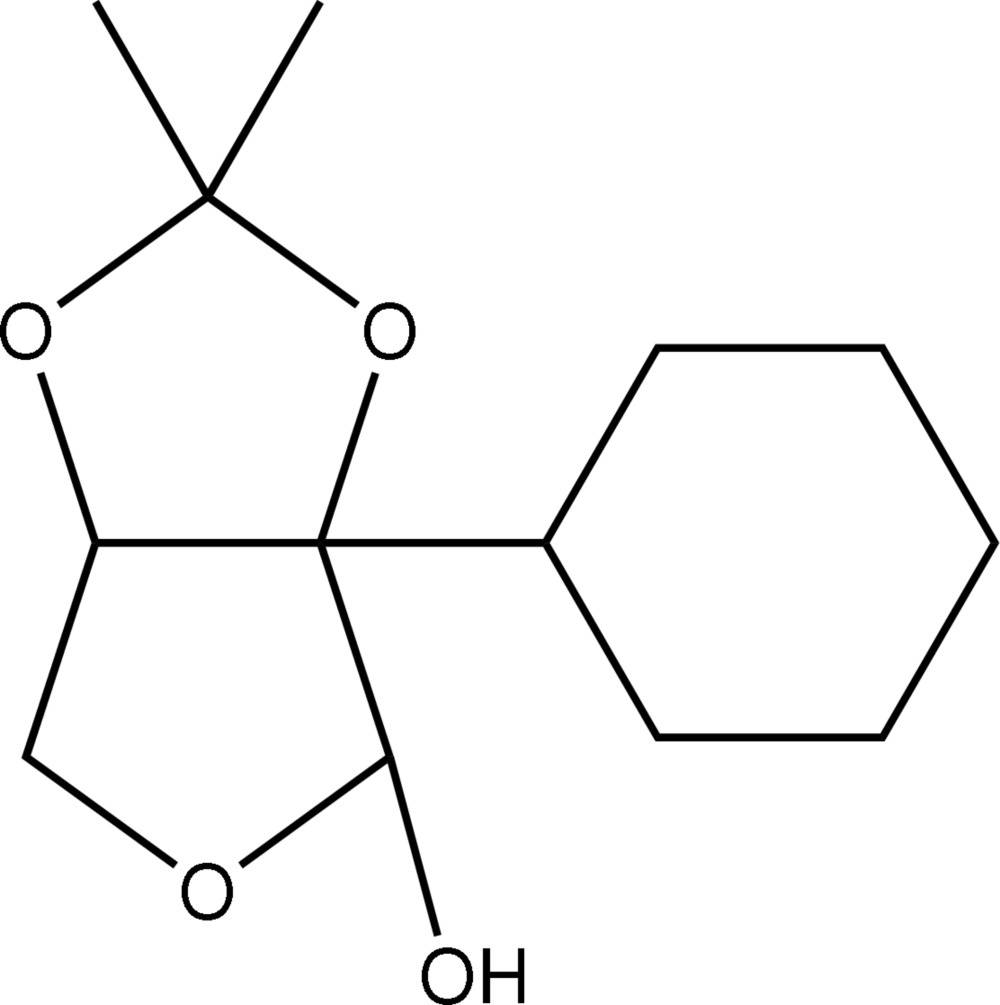


## Experimental

### 

#### Crystal data

C_13_H_22_O_4_
*M* *_r_* = 242.31
Triclinic, 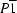

*a* = 5.454 (3) Å
*b* = 9.908 (3) Å
*c* = 12.442 (5) Å
α = 93.29 (3)°
β = 94.95 (4)°
γ = 102.94 (3)°
*V* = 650.8 (5) Å^3^
*Z* = 2
Mo *K*α radiation
μ = 0.09 mm^−1^
*T* = 153 K
0.24 × 0.15 × 0.13 mm

#### Data collection

Rigaku AFC12K/SATURN724 diffractometer
Absorption correction: multi-scan (*ABSCOR*; Higashi, 1995 ▶) *T*_min_ = 0.789, *T*_max_ = 1
4992 measured reflections
2224 independent reflections
2064 reflections with *I* > 2σ(*I*)
*R*_int_ = 0.020

#### Refinement

*R*[*F* ^2^ > 2σ(*F* ^2^)] = 0.046
*wR*(*F* ^2^) = 0.124
*S* = 1.09
2224 reflections
156 parameters
1 restraint
H-atom parameters constrained
Δρ_max_ = 0.18 e Å^−3^
Δρ_min_ = −0.20 e Å^−3^

### 

Data collection: *CrystalClear* (Rigaku/MSC, 2005 ▶); cell refinement: *CrystalClear*; data reduction: *CrystalClear*; program(s) used to solve structure: *SHELXS97* (Sheldrick, 2008 ▶); program(s) used to refine structure: *SHELXL97* (Sheldrick, 2008 ▶); molecular graphics: *DIAMOND* (Brandenburg, 2006 ▶); software used to prepare material for publication: *publCIF* (Westrip, 2009 ▶).

## Supplementary Material

Crystal structure: contains datablocks global, I. DOI: 10.1107/S1600536809048557/hg2594sup1.cif

Structure factors: contains datablocks I. DOI: 10.1107/S1600536809048557/hg2594Isup2.hkl

Additional supplementary materials:  crystallographic information; 3D view; checkCIF report

## Figures and Tables

**Table 1:** Hydrogen-bond geometry (Å, °)

*D*—H⋯*A*	*D*—H	H⋯*A*	*D*⋯*A*	*D*—H⋯*A*
O4—H40⋯O3^i^	0.84	1.95	2.787 (2)	173
C2—H2⋯O1^ii^	1.00	2.43	3.350 (3)	152

Symmetry codes: (i) 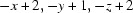; (ii) 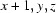.

**Fig. 1.**
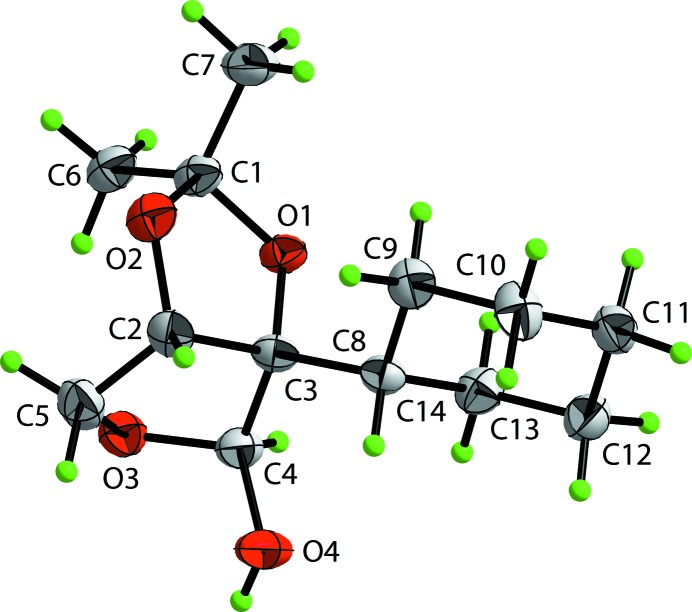
Molecular structure of (I) showing atom-labelling scheme and displacement ellipsoids at the 50% probability level.

**Fig. 2.**
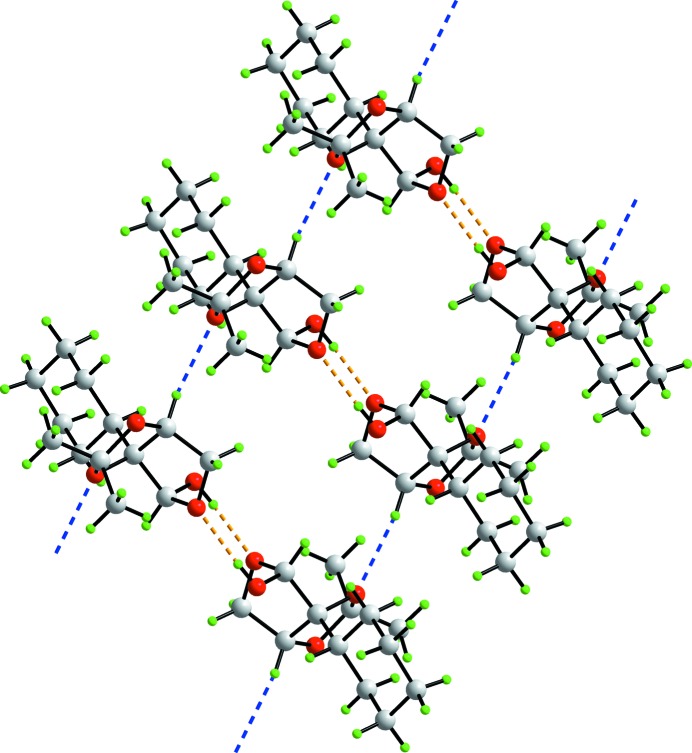
Supramolecular chain formation along the a axis in (I) mediated by O—H···O hydrogen bonds (orange dashed lines) and C—H···O contacts (blue dashed lines).

## supplementary crystallographic information

### Comment

Recently we examined the dihydroxylation of the olefin portion of 1,2-dioxines, which provided access to a range of polyhydroxylated core structures upon selective manipulation of the peroxide linkage (Robinson *et al.*, 2006; Robinson *et al.*, 2009; Valente *et al.*, 2009). This methodology was extended to include the synthesis of erythrono-γ-lactones (Pedersen *et al.*, 2009), during the course of which the title compound, (I), was prepared.

The molecular structure of (I), Fig. 1, comprises two fused five-membered rings linked by the C2—C3 bond. The acetonide ring adopts an envelope conformation with the O2 atom being the flap atom. A twisted conformation is found for the furanose ring whereby the O3 atom is *endo* and the C4 atom is *exo*. The cyclohexyl group is in the chair conformation. The crystal structure comprises centrosymmetric dimers held by {···OCOH}_2_ synthons arising from the interaction between the O4-hydroxyl group and the ether-O3 atom, Fig. 2 and Table 1. The resultant eight-membered ring has an elongated chair conformation. The dimeric aggregates are linked into supramolecular chains *via* C—H···O interactions, Fig. 2 and Table 1. The topology of the supramolecular chain is linear, and is aligned along the *a* direction.

### Experimental

For full synthetic procedures and characterization data see Pedersen *et al.* (2009) and Robinson *et al.* (2009). To a stirred solution of Co(salen)_2_ (27 mg, 0.08 mmol) in THF (5 ml) at ambient temperature was added (3aR,7aS)-3a-cyclohexyl-tetrahydro-2,2-dimethyl-[1,3]dioxolo[4,5-*d*][1,2]dioxine (803 mg, 3.31 mmol). The reaction left to stir until complete by TLC (~16 h). All volatiles were removed *in vacuo* giving a crude mixture of regioisomers in a 43:57 ratio. The isomers were completely separated by flash chromatography giving a combined total yield of 779 mg (97%). Compound (I) was isolated as a colourless solid (337 mg), and the pure material was recrystallized by slowly evaporating a 1:1 mixture of dichloromethane/heptane to give colourless prisms, m. pt. 391–394 K. The compound was found to exist solely in its cyclic hemi-acetal form(*s*) both as a solid indicated by IR (absence of carbonyl signal), and in CDCl_3_ solution which revealed a 94:6 anomeric ratio.

### Refinement

Carbon-bound H-atoms were placed in calculated positions (C–H 0.98–1.00 Å) and were included in the refinement in the riding model approximation with *U*_iso_(H) set to 1.2–1.5*U*_eq_(C). A rotating group model was used for the methyl groups. The O–bound H-atom was located in a difference Fourier map and was refined with an O–H restraint of 0.840±0.001 Å, and with *U_iso_*(H) = 1.*5U_eq_*(O).

### Crystal data

**Table:**

C_13_H_22_O_4_	*Z* = 2
*M**_r_* = 242.31	*F*(000) = 264
Triclinic, *P*1	*D*_x_ = 1.237 Mg m^−^^3^
Hall symbol: -P 1	Mo *K*α radiation, λ = 0.71073 Å
*a* = 5.454 (3) Å	Cell parameters from 657 reflections
*b* = 9.908 (3) Å	θ = 2.1–30.2°
*c* = 12.442 (5) Å	µ = 0.09 mm^−^^1^
α = 93.29 (3)°	*T* = 153 K
β = 94.95 (4)°	Needle, colourless
γ = 102.94 (3)°	0.24 × 0.15 × 0.13 mm
*V* = 650.8 (5) Å^3^	

### Data collection

**Table:**

Rigaku AFC12K/SATURN724 diffractometer	2224 independent reflections
Radiation source: fine-focus sealed tube	2064 reflections with *I* > 2σ(*I*)
graphite	*R*_int_ = 0.020
ω scans	θ_max_ = 25.0°, θ_min_ = 2.1°
Absorption correction: multi-scan (*ABSCOR*; Higashi, 1995)	*h* = −6→5
*T*_min_ = 0.789, *T*_max_ = 1	*k* = −11→11
4992 measured reflections	*l* = −14→14

### Refinement

**Table:**

Refinement on *F*^2^	Primary atom site location: structure-invariant direct methods
Least-squares matrix: full	Secondary atom site location: difference Fourier map
*R*[*F*^2^ > 2σ(*F*^2^)] = 0.046	Hydrogen site location: inferred from neighbouring sites
*wR*(*F*^2^) = 0.124	H-atom parameters constrained
*S* = 1.09	*w* = 1/[σ^2^(*F*_o_^2^) + (0.066*P*)^2^ + 0.1734*P*] where *P* = (*F*_o_^2^ + 2*F*_c_^2^)/3
2224 reflections	(Δ/σ)_max_ < 0.001
156 parameters	Δρ_max_ = 0.18 e Å^−^^3^
1 restraint	Δρ_min_ = −0.20 e Å^−^^3^

### Special details

**Table:**

Geometry. All s.u.'s (except the s.u. in the dihedral angle between two l.s. planes) are estimated using the full covariance matrix. The cell s.u.'s are taken into account individually in the estimation of s.u.'s in distances, angles and torsion angles; correlations between s.u.'s in cell parameters are only used when they are defined by crystal symmetry. An approximate (isotropic) treatment of cell s.u.'s is used for estimating s.u.'s involving l.s. planes.
Refinement. Refinement of *F*^2^ against ALL reflections. The weighted *R*-factor *wR* and goodness of fit *S* are based on *F*^2^, conventional *R*-factors *R* are based on *F*, with *F* set to zero for negative *F*^2^. The threshold expression of *F*^2^ > 2σ(*F*^2^) is used only for calculating *R*-factors(gt) *etc*. and is not relevant to the choice of reflections for refinement. *R*-factors based on *F*^2^ are statistically about twice as large as those based on *F*, and *R*- factors based on ALL data will be even larger.

### Fractional atomic coordinates and isotropic or equivalent isotropic displacement parameters (Å^2^)

**Table:**

	*x*	*y*	*z*	*U*_iso_*/*U*_eq_	
O1	0.69690 (18)	0.26256 (11)	0.70713 (8)	0.0281 (3)	
O2	1.0523 (2)	0.34466 (12)	0.62499 (9)	0.0352 (3)	
O3	0.9937 (2)	0.48884 (11)	0.85907 (9)	0.0377 (3)	
O4	1.0310 (3)	0.31604 (12)	0.97416 (9)	0.0439 (4)	
H4O	1.0281	0.3703	1.0282	0.066*	
C1	0.7854 (3)	0.33328 (17)	0.61509 (12)	0.0312 (4)	
C2	1.1367 (3)	0.33878 (16)	0.73567 (13)	0.0320 (4)	
H2	1.2783	0.2898	0.7425	0.038*	
C3	0.9031 (3)	0.25734 (15)	0.78511 (12)	0.0270 (4)	
C4	0.8885 (3)	0.35109 (16)	0.88634 (12)	0.0327 (4)	
H4	0.7088	0.3415	0.9019	0.039*	
C5	1.2089 (3)	0.47939 (18)	0.80110 (15)	0.0410 (4)	
H5A	1.3609	0.4853	0.8524	0.049*	
H5B	1.2450	0.5556	0.7526	0.049*	
C6	0.7237 (3)	0.47484 (18)	0.61632 (14)	0.0366 (4)	
H6A	0.5397	0.4637	0.6085	0.055*	
H6B	0.7959	0.5243	0.5562	0.055*	
H6C	0.7954	0.5282	0.6850	0.055*	
C7	0.6697 (4)	0.2440 (2)	0.51267 (14)	0.0446 (5)	
H7A	0.4850	0.2278	0.5085	0.067*	
H7B	0.7193	0.1549	0.5134	0.067*	
H7C	0.7299	0.2916	0.4497	0.067*	
C8	0.9010 (3)	0.10690 (15)	0.80714 (12)	0.0281 (4)	
H8	1.0454	0.1086	0.8629	0.034*	
C9	0.9368 (3)	0.01889 (16)	0.70736 (14)	0.0353 (4)	
H9A	0.7968	0.0159	0.6504	0.042*	
H9B	1.0973	0.0623	0.6788	0.042*	
C10	0.9421 (3)	−0.12889 (17)	0.73441 (16)	0.0410 (4)	
H10A	0.9581	−0.1844	0.6677	0.049*	
H10B	1.0915	−0.1265	0.7863	0.049*	
C11	0.7028 (3)	−0.19803 (17)	0.78331 (14)	0.0363 (4)	
H11A	0.5557	−0.2117	0.7280	0.044*	
H11B	0.7179	−0.2905	0.8053	0.044*	
C12	0.6588 (3)	−0.11062 (17)	0.88099 (14)	0.0376 (4)	
H12A	0.7936	−0.1083	0.9402	0.045*	
H12B	0.4948	−0.1539	0.9067	0.045*	
C13	0.6576 (3)	0.03762 (17)	0.85399 (13)	0.0343 (4)	
H13A	0.6391	0.0929	0.9204	0.041*	
H13B	0.5107	0.0361	0.8010	0.041*	

### Atomic displacement parameters (Å^2^)

**Table:**

	*U*^11^	*U*^22^	*U*^33^	*U*^12^	*U*^13^	*U*^23^
O1	0.0218 (5)	0.0379 (6)	0.0252 (6)	0.0082 (4)	0.0014 (4)	0.0048 (4)
O2	0.0277 (6)	0.0454 (7)	0.0345 (6)	0.0098 (5)	0.0089 (5)	0.0062 (5)
O3	0.0485 (7)	0.0307 (6)	0.0335 (7)	0.0112 (5)	−0.0019 (5)	−0.0002 (5)
O4	0.0662 (8)	0.0383 (7)	0.0279 (6)	0.0212 (6)	−0.0115 (6)	−0.0033 (5)
C1	0.0266 (8)	0.0414 (9)	0.0262 (8)	0.0078 (6)	0.0047 (6)	0.0051 (6)
C2	0.0240 (8)	0.0352 (8)	0.0373 (9)	0.0082 (6)	0.0009 (6)	0.0041 (7)
C3	0.0234 (7)	0.0332 (8)	0.0250 (8)	0.0095 (6)	−0.0009 (6)	−0.0001 (6)
C4	0.0413 (9)	0.0312 (8)	0.0264 (8)	0.0125 (7)	−0.0015 (7)	0.0008 (6)
C5	0.0334 (9)	0.0370 (9)	0.0483 (10)	0.0027 (7)	−0.0044 (7)	0.0023 (8)
C6	0.0343 (9)	0.0440 (10)	0.0337 (9)	0.0121 (7)	0.0037 (7)	0.0100 (7)
C7	0.0490 (11)	0.0548 (11)	0.0272 (9)	0.0082 (8)	−0.0003 (8)	0.0005 (8)
C8	0.0260 (8)	0.0313 (8)	0.0272 (8)	0.0084 (6)	0.0005 (6)	0.0000 (6)
C9	0.0347 (9)	0.0351 (9)	0.0373 (9)	0.0079 (7)	0.0124 (7)	−0.0006 (7)
C10	0.0407 (10)	0.0329 (9)	0.0506 (11)	0.0097 (7)	0.0137 (8)	−0.0047 (7)
C11	0.0380 (9)	0.0314 (8)	0.0378 (9)	0.0050 (7)	0.0042 (7)	−0.0006 (7)
C12	0.0432 (10)	0.0365 (9)	0.0340 (9)	0.0088 (7)	0.0070 (7)	0.0061 (7)
C13	0.0369 (9)	0.0369 (9)	0.0319 (9)	0.0119 (7)	0.0107 (7)	0.0027 (7)

### Geometric parameters (Å, °)

**Table:**

O1—C3	1.4328 (18)	C7—H7A	0.9800
O1—C1	1.4392 (19)	C7—H7B	0.9800
O2—C2	1.423 (2)	C7—H7C	0.9800
O2—C1	1.429 (2)	C8—C9	1.528 (2)
O3—C4	1.429 (2)	C8—C13	1.531 (2)
O3—C5	1.447 (2)	C8—H8	1.0000
O4—C4	1.392 (2)	C9—C10	1.527 (2)
O4—H4O	0.8399	C9—H9A	0.9900
C1—C7	1.512 (2)	C9—H9B	0.9900
C1—C6	1.513 (2)	C10—C11	1.526 (2)
C2—C5	1.526 (2)	C10—H10A	0.9900
C2—C3	1.544 (2)	C10—H10B	0.9900
C2—H2	1.0000	C11—C12	1.519 (2)
C3—C8	1.529 (2)	C11—H11A	0.9900
C3—C4	1.540 (2)	C11—H11B	0.9900
C4—H4	1.0000	C12—C13	1.527 (2)
C5—H5A	0.9900	C12—H12A	0.9900
C5—H5B	0.9900	C12—H12B	0.9900
C6—H6A	0.9800	C13—H13A	0.9900
C6—H6B	0.9800	C13—H13B	0.9900
C6—H6C	0.9800		
			
C3—O1—C1	111.24 (11)	C1—C7—H7B	109.5
C2—O2—C1	108.79 (12)	H7A—C7—H7B	109.5
C4—O3—C5	105.95 (12)	C1—C7—H7C	109.5
C4—O4—H4O	108.8	H7A—C7—H7C	109.5
O2—C1—O1	105.26 (12)	H7B—C7—H7C	109.5
O2—C1—C7	108.62 (14)	C9—C8—C3	113.25 (13)
O1—C1—C7	109.15 (13)	C9—C8—C13	109.62 (13)
O2—C1—C6	111.35 (13)	C3—C8—C13	111.20 (12)
O1—C1—C6	110.47 (13)	C9—C8—H8	107.5
C7—C1—C6	111.76 (14)	C3—C8—H8	107.5
O2—C2—C5	114.39 (14)	C13—C8—H8	107.5
O2—C2—C3	104.95 (12)	C10—C9—C8	111.22 (14)
C5—C2—C3	104.74 (13)	C10—C9—H9A	109.4
O2—C2—H2	110.8	C8—C9—H9A	109.4
C5—C2—H2	110.8	C10—C9—H9B	109.4
C3—C2—H2	110.8	C8—C9—H9B	109.4
O1—C3—C8	110.40 (12)	H9A—C9—H9B	108.0
O1—C3—C4	108.10 (12)	C11—C10—C9	111.32 (14)
C8—C3—C4	114.24 (13)	C11—C10—H10A	109.4
O1—C3—C2	103.39 (12)	C9—C10—H10A	109.4
C8—C3—C2	116.55 (12)	C11—C10—H10B	109.4
C4—C3—C2	103.27 (12)	C9—C10—H10B	109.4
O4—C4—O3	111.08 (13)	H10A—C10—H10B	108.0
O4—C4—C3	109.45 (12)	C12—C11—C10	111.32 (14)
O3—C4—C3	104.59 (13)	C12—C11—H11A	109.4
O4—C4—H4	110.5	C10—C11—H11A	109.4
O3—C4—H4	110.5	C12—C11—H11B	109.4
C3—C4—H4	110.5	C10—C11—H11B	109.4
O3—C5—C2	106.01 (13)	H11A—C11—H11B	108.0
O3—C5—H5A	110.5	C11—C12—C13	111.60 (14)
C2—C5—H5A	110.5	C11—C12—H12A	109.3
O3—C5—H5B	110.5	C13—C12—H12A	109.3
C2—C5—H5B	110.5	C11—C12—H12B	109.3
H5A—C5—H5B	108.7	C13—C12—H12B	109.3
C1—C6—H6A	109.5	H12A—C12—H12B	108.0
C1—C6—H6B	109.5	C12—C13—C8	111.60 (13)
H6A—C6—H6B	109.5	C12—C13—H13A	109.3
C1—C6—H6C	109.5	C8—C13—H13A	109.3
H6A—C6—H6C	109.5	C12—C13—H13B	109.3
H6B—C6—H6C	109.5	C8—C13—H13B	109.3
C1—C7—H7A	109.5	H13A—C13—H13B	108.0
			
C2—O2—C1—O1	−24.16 (15)	C2—C3—C4—O4	−89.45 (15)
C2—O2—C1—C7	−140.95 (13)	O1—C3—C4—O3	−79.48 (14)
C2—O2—C1—C6	95.56 (15)	C8—C3—C4—O3	157.20 (12)
C3—O1—C1—O2	12.78 (15)	C2—C3—C4—O3	29.63 (14)
C3—O1—C1—C7	129.21 (14)	C4—O3—C5—C2	34.96 (16)
C3—O1—C1—C6	−107.53 (14)	O2—C2—C5—O3	99.37 (16)
C1—O2—C2—C5	−88.63 (16)	C3—C2—C5—O3	−14.97 (16)
C1—O2—C2—C3	25.58 (15)	O1—C3—C8—C9	62.51 (16)
C1—O1—C3—C8	−122.93 (13)	C4—C3—C8—C9	−175.42 (12)
C1—O1—C3—C4	111.46 (14)	C2—C3—C8—C9	−55.00 (17)
C1—O1—C3—C2	2.43 (14)	O1—C3—C8—C13	−61.44 (16)
O2—C2—C3—O1	−16.78 (14)	C4—C3—C8—C13	60.62 (17)
C5—C2—C3—O1	104.03 (13)	C2—C3—C8—C13	−178.95 (12)
O2—C2—C3—C8	104.51 (14)	C3—C8—C9—C10	178.09 (12)
C5—C2—C3—C8	−134.68 (14)	C13—C8—C9—C10	−57.09 (17)
O2—C2—C3—C4	−129.38 (12)	C8—C9—C10—C11	56.64 (19)
C5—C2—C3—C4	−8.57 (15)	C9—C10—C11—C12	−54.5 (2)
C5—O3—C4—O4	77.45 (16)	C10—C11—C12—C13	53.94 (19)
C5—O3—C4—C3	−40.52 (15)	C11—C12—C13—C8	−55.52 (18)
O1—C3—C4—O4	161.44 (12)	C9—C8—C13—C12	56.55 (17)
C8—C3—C4—O4	38.13 (18)	C3—C8—C13—C12	−177.46 (12)

### Hydrogen-bond geometry (Å, °)

**Table:**

*D*—H···*A*	*D*—H	H···*A*	*D*···*A*	*D*—H···*A*
O4—H40···O3^i^	0.84	1.95	2.787 (2)	173
C2—H2···O1^ii^	1.00	2.43	3.350 (3)	152

Symmetry codes: (i) −*x*+2, −*y*+1, −*z*+2; (ii) *x*+1, *y*, *z*.
